# Genetics of Severe Cutaneous Adverse Reactions

**DOI:** 10.3389/fmed.2021.652091

**Published:** 2021-07-15

**Authors:** Shang-Chen Yang, Chun-Bing Chen, Mao-Ying Lin, Zhi-Yang Zhang, Xiao-Yan Jia, Ming Huang, Ya-Fen Zou, Wen-Hung Chung

**Affiliations:** ^1^Department of Dermatology, Xiamen Chang Gung Allergology Consortium, Xiamen Chang Gung Hospital, Xiamen, China; ^2^Department of Dermatology, Drug Hypersensitivity Clinical and Research Center, Chang Gung Memorial Hospital, Taoyuan, Taiwan; ^3^Cancer Vaccine and Immune Cell Therapy Core Laboratory, Department of Medical Research, Chang Gung Memorial Hospital, Taoyuan, Taiwan; ^4^College of Medicine, Chang Gung University, Taoyuan, Taiwan; ^5^Whole-Genome Research Core Laboratory of Human Diseases, Chang Gung Memorial Hospital, Keelung, Taiwan; ^6^Graduate Institute of Clinical Medical Sciences, Chang Gung University, Taoyuan, Taiwan; ^7^Immune-Oncology Center of Excellence, Chang Gung Memorial Hospital, Linkou, Taiwan; ^8^Department of Neurology, Xiamen Chang Gung Hospital, Xiamen, China; ^9^Department of Dermatology, Beijing Tsinghua Chang Gung Hospital, School of Clinical Medicine, Tsinghua University, Beijing, China; ^10^Department of Dermatology, Ruijin Hospital, School of Medicine, Shanghai Jiaotong University, Shanghai, China; ^11^Genomic Medicine Core Laboratory, Chang Gung Memorial Hospital, Linkou, Taiwan

**Keywords:** genetic screen, Stevens–Johnson syndrome, toxic epidermal necrolysis, pharmacogenomic, severe cutaneous adverse drug reactions

## Abstract

Severe cutaneous adverse reactions (SCARs) including Stevens-Johnson syndrome (SJS), toxic epidermal necrolysis (TEN), and drug rash with eosinophilia and systemic symptoms (DRESS) are T cells-mediated life-threatening immune reactions, most commonly induced by drug. The last decade has seen significant progress in SCARs research. Recent studies have unveiled the pathogenesis of SCARs involved in susceptible genes, including human leukocyte antigens (HLA) and drugs-T cell receptor (TCR) interaction that may trigger T cell activation with downstream immune signaling of cytokines/chemokines and specific cytotoxic proteins releases. Advances in identification of multiple genetic alleles associated with specific drugs related SCARS in different populations is an important breakthrough in recent years for prevention of SCARs. This article summarized the findings on genetic factors related to SJS/TEN, especially for HLA.

## Introduction

Cutaneous reactions are most common manifestations seen in hypersensitivity reactions of drugs, named cutaneous adverse drug reactions, and among those with risk of life-threatening are classified into severe cutaneous adverse reactions (SCARs), mediated by drug-specific T lymphocytes, including drug rash with eosinophilia and systemic symptoms (DRESS)/drug-induced hypersensitivity syndrome (DIHS), Stevens–Johnson syndrome (SJS), and toxic epidermal necrolysis (TEN). The mortality rates for SJS/TEN are ~5–10% in SJS and 30–50% in TEN (1), and the common leading culprit drugs around the world are antibiotics, antiepileptics, allopurinol, and cold medicine (2–4). In this article, we reviewed the updated molecular mechanism and susceptible genes related to SCARs.

### Immune Mechanisms and Clinical Manifestations of Severe Cutaneous Adverse Reactions

SCARs belonged to type IV hypersensitivity reaction and are T cell-mediated immune responses with different effector cells and cytokines, resulting in further subtypes with specific clinical features.

SJS/TEN is characteristic for cutaneous and mucosal detachment, commonly including oral, ocular, and genital or anal mucosae. Internal epithelial organs are rarely involved, concerning mainly in the respiratory and gastrointestinal tract (5). SJS/TEN is one of the representative SCARs with high mortality and complications, where CD8+ cytotoxic T lymphocytes (CTLs) play a role, acting as effector cells with nature killer cells, causing cell death by the participant of perforin/granzyme B, granulysin, and/or Fas-Fas ligand (6). Besides, cytokines/chemokines, including interleukin (IL)-6, IL-8, IL-15, C-C chemokine receptor 10, tumor necrosis factor-α, interferon-γ, etc., also contribute to these severe immune reactions (7). The messenger RNA expression and level of 15-kDa granulysin were found much higher than others in SJS/TEN blister cells, suggesting it is the key mediator of disseminated keratinocyte apoptosis (8).

Another severe phenotypes of SCARs is DRESS, presenting as long-lasting, widespread, and infiltrated skin rash with internal organ involvement and hematological abnormalities, most frequently as eosinophilia and atypical lymphocytes (9). The immune mechanism involved in DRESS is majorly the Th2 immune response. Th2 cells involved in DRESS secrete ILs, including IL-5, IL-4, and IL-13, promoting immunoglobulin E and immunoglobulin G4 production and macrophage deactivation, leading to mast cell and eosinophil activation (6). Perforin/granzyme B, tumor necrosis factor-α, interferon-γ, C-C chemokine receptor 4, and thymus and activation-regulated chemokine (10) are also engaged.

### Molecular Mechanism and Susceptible Genes Related to Severe Cutaneous Adverse Reactions

Roujeau et al. firstly reported the relationship between human leukocyte antigen (HLA) and SJS in 1986 (11) and HLA and TEN in 1987 (12). The association of HLA and SCARs are drug-specific and ethnicity-specific (13). Specific HLA is not only a genetic marker but also plays an important role in the pathogenesis of SCARs by presenting drug antigen to T cell receptor (TCR) and causing T cell-dependent immune response (14).

Recent advance in the technology of pharmacogenomic studies have showed more genetic risk factors associated with SCARs, not only in genes of HLA and other immune pathways, but also in drug metabolism or elimination. The genetic approach for the pharmacogenomics studies for SCAR has been evolved from sequencing based genotyping with polymerase chain reaction (PCR), such as HLA genotypes (15) to more comprehensive approaches, including genome-wide association study (GWAS) and next-generation sequencing (NGS), in discovering the relationship between adverse drug reactions (ADRs) and genotyping, and discovered more non-HLA loci (15–17).

### Genetic Susceptibility to Aromatic Antiepileptic Drug-Induced Severe Cutaneous Adverse Reactions

Aromatic antiepileptic drugs (AEDs) are commonly used mainly in treating epilepsy and neuralgia, including carbamazepine (CBZ), oxcarbazepine (OXC), lamotrigine (LTG), phenytoin (PHT), and phenobarbital (PB). A strong association of HLA-B^*^15:02 with CBZ-induced SJS/TEN was first found in Han Chinese (18). This relationship was also proved in Southeast Asians, including Thais (19), Malaysians (20), Indians (21).

AEDs had the same chemical structure, an aromatic ring, thus sharing the same risk allele. The susceptibility of HLA-B^*^15:02 with AED-induced SJS/TEN was also found in PHT (22) and OXC (23), causing SJS/TEN in Han Chinese and Southeast Asians (24–26) (Table 1).

**Table 1:** Genetic associations with SCARs in different populations.

**Causative drug**	**Genetic associations**	**SCAR**	**Ethnicity**	**Reference**	**Clinical implementations and recommendations**
**Antiepileptic drug**					
Carbamazepine	B^*^15:02	SJS/TEN	Han Chinese	(18, 25)	1. Labeled by Taiwan FDA, Hong Kong Department of Health, Singapore Ministry of Health, Thailand HITAP, India MOHFW, US FDA, Canada HCSC, EMA. 2. National health insurance subsidized in Taiwan, China, Hong Kong, Singapore, and Thailand.
			Southeast Asian	(19, 20)	
	B^*^15:11	SJS/TEN	Northeast Asian	(27, 28)	
			Southeast Asian	(29)	
	B^*^15:21	SJS/TEN	Southeast Asian	(29)	
	B^*^57:01	SJS/TEN	Caucasians	(30)	
	A^*^31:01	DRESS	Han Chinese	(31)	1. Labeled by US FDA, Canada HCSC, Japan, and Taiwan.
			Caucasians	(31, 32)	
		SCARs	Japanese	(33)	
Oxcarbazepine	B*15:02	SJS/TEN	Han Chinese	(23, 25)	1. Labeled by US FDA, EMA and Taiwan FDA. 2. Ongoing clinical trial in Taiwan and China.
Lamotrigine	B*15:02	SJS/GEN	Han Chinese	(25)	Ongoing clinical trial in Taiwan and China.
	B*38	SJS/TEN	Caucasians	(34)	
Phenytoin	B*15:02	SCARs	Han Chinese	(22, 25, 35)	1. Labeled by Canada HCSC, US FDA, and Taiwan FDA. 2. Ongoing clinical trial in Taiwan and China.
			Southeast Asian	(24, 26)	
	B*13:01	SCARs	Asian	(35)	Ongoing clinical trial in Taiwan and China.
	B*15:13	SJS/TEN	Japanese	(36)	Ongoing clinical trial in Taiwan and China.
	B*51:01	SCARs	Han Chinese	(37)	
	CYP2C9*3	SCARs	Asian	(35)	Ongoing clinical trial in Taiwan and China
Phenobarbital	B*51:01	SJS/TEN	Japanese	(36)	
	A*02:07	SJS/TEN	Japanese	(36)	
Zonisamide	A*02:07	SJS/TEN	Japanese	(36)	
**Antiinfection drugs**					
Antibiotics					
Co-trimoxazole	B*13:01	SCARs	Han Chinese Southeast Asian	(17)	
Piperacillin/tazobactam	B*62	DRESS	Caucasians	(38)	
Sulfamethoxazole	B*38	SJS/TEN	Caucasians	(34)	
Vancomycin	A*32:01	DRESS	Caucasians	(39)	
Anti-virus					
Abacavir	B*57:01	HSR	Caucasians	(40, 41)	Labeled by US FDA, US HHS, EMA, Canada HCSC, and multiple international HIV/AIDS organizations
			Hispanic	(42)	
			Indian	(43)	
Nevirapine	B^*^35:05	HSR	Thai	(44)	
	C^*^04:01	SJS/TEN	African	(45)	
	Cw^*^04	cADRs	All	(46)	
	Cw^*^08	HSR	Sardinian	(47)	
		Japanese	(48)	
	DRB1^*^01:01	HSR	Caucasians	(49)	
	CYP2B6	cADRs	All	(46)	
	CCHCR1	HSR	Thai	(44)	
Raltegravir	B*53:01	DRESS	African	(50)	
Anti-leprosy					
Dapsone	B*13:01	DHS	Han Chinese	(51)	Prospective screening in China.
			Korean	(52)	
			Indonesian	(53)	
		SCARs	Thai	(54)	
**Cold medicine**					
	A*02:06	SJS/TEN	Japanese	(55)	
			Korean	(56, 57)	
	A*66:01	SJS/TEN	Brazilian	(58)	
	B*44:03	SJS/TEN	Japanese	(55)	
			Indian	(56)	
			Thai	(59)	
			Brazilian	(56, 58)	
	C*03:04	SJS/TEN	Korean	(57)	
	C*12:03	SJS/TEN	Brazilian	(58)	
	TLR3	SJS/TEN	Japanese	(60)	
	PTGER3	SJS/TEN	Japanese	(60)	
	IKZF1	SJS/TEN	Japanese	(61)	
**Others**					
Allopurinol	B*58:01	SJS/TEN	Thai	(62)	1. Recommendation in the American College of Rheumatology guidelines for allopurinol initiation in Asians. 2. National health insurance subsidized in Taiwan, China, Korea, and Thailand.
			Japanese	(63)	
		SCARs	Han Chinese	(64)	
			Korean	(65)	
			Caucasians	(34)	
Methazolamide	B*59:01	SJS/TEN	Northeast Asian	(66)	
			Han Chinese	(67)	

*DHS, Dapsone hypersensitivity syndrome; HSR, hypersensitivity reaction*.

By using GWAS approach, our previous study found that CYP2C9^*^3 was significantly related to PHT-induced SCARs in Han Chinese, Japanese and Malaysian, and by adding HLA-B*15:02 to this variant could increase sensitivity for preemptive test (35). In addition to HLA-B^*^15:02, HLA-B^*^13:01, and HLA-B^*^51:01 were also found strong association with PHT-induced SCARs in Han Chinese, Thai and Japanese (37). Another genetic variant for PHT-induced SCARs was HLA-B^*^15:13, which majorly found in Malaysian (24) (Table 1).

The HLA-DRB1^*^15:01 allele had been reported to be associated with AED-induced SJS/TEN in Han Chinese (68). Besides, HLA-A^*^33:03, HLA-B^*^58:01, HLA-B^*^40:01, and HLA-DRB1^*^03:01 alleles had also been reported to be associated with AED-induced SJS/TEN (68, 69).

There were differences of susceptible genes among ethnic groups. The same susceptible genotype of HLA-B*15:02 of CBZ-induced SJS/TEN was not found in Northeast Asians like Japanese and Korean, but HLA-B^*^15:11 instead (27, 28, 63). Interestingly, association between HLA-B*15:21 and CBZ-induced SJS was found in an HLA-B^*^15:02 negative Thai patient (29). The relationship of HLA-B^*^15:11 and HLA-B^*^15:21, member of HLA-B75 serotype, and CBZ-induced SJS/TEN was found in HLA-B^*^15:02 negative patients with pooled-data of Southeast Asian (29) (Table 1).

As for other AEDs in Northeast Asians, HLA-B^*^51:01 was found to be related to PHT- and PB-induced SJS/TEN, and HLA-A^*^02:07 was found to be relevant with PB- and zonisamide-induced SJS/TEN (36) (Table 1).

Caucasians also revealed less relevant of AEDs and HLA-B^*^15:02, instead, they were commonly found with HLA-A^*^31:01, and developed CBZ-induced DRESS (31, 32). This relationship also existed in Han Chinese and Japanese (31, 70), however, not only DRESS but also SJS/TEN were related with this gene in Japanese (70). Another Caucasian-specific gene was HLA-B^*^57:01 and was found to be strongly associated with CBZ-induced SJS/TEN in Europeans (30). A limited number also showed a relationship with HLA-B^*^38 and LTG-induced SJS/TEN (34) (Table 1).

### Genetic Susceptibility to Antibiotics Induced Severe Cutaneous Adverse Reactions

Antibiotics are one of the most widely used medications, being responsible for one-fifth to one-third of ADRs (71, 72). The most common cause of SCARs induced by antibiotics are beta-lactams; others include sulphonamide antibiotics, fluoroquinolones, macrolides, tetracyclines, and glycopeptides (71). Above all, vancomycin is notable for causing DRESS. The relevant susceptible gene found with vancomycin-causing DRESS was HLA-A^*^32:01, with a strong association in Caucasians (39). Other possible genes as a risk factor in Caucasian were HLA-B^*^38 with sulfamethoxazole-induced SJS/TEN (34) and HLA-B^*^62 with piperacillin/tazobactam-induced DRESS (38), yet further research is still needed due to number limitation (Table 1).

Antibiotics, AEDs, and allopurinol are the most common offending drugs causing SJS/TEN in China from our previous study (4). Although the finding of genetic susceptibility with antibiotic-induced SCARs was limited, our recent study by using whole genome sequencing (WGS) approach showed HLA-B^*^13:01, not metabolism enzymes, was strong associated with co-trimoxazole-induced SCARs in Asians, including Han Chinese, Thai, and Malaysian (17) (Table 1).

Dapsone is mainly used in the treatment of leprosy and can also apply in other dermatological inflammatory diseases due to the ability of anti-infection and anti-inflammation. Dapsone hypersensitivity syndrome is the potentially fatal adverse effect of dapsone, presenting as fever, skin rash, lymphadenopathy, and multiple systemic involvements. By using GWAS approach, the susceptible gene of DHS was first found in Han Chinese with HLA-B^*^13:01 (51), which mainly exists in Asians. The same result was validated in Koreans (52) and Indonesians (53). This gene was also susceptible in Thai with dapsone-induced SCARs (54), including DRESS and SJS/TEN (Table 1).

### Genetic Susceptibility to Antivirus Induced Hypersensitivity Reactions

Abacavir is a nucleoside reverse transcriptase inhibitor and is used as one of the combination treatment of human immunodeficiency virus (HIV) infection. Hypersensitivity reactions induced by abacavir appear in ~5–8% of Caucasians (73), which presented as fever, rash, gastrointestinal tract, and respiratory symptoms during the first 6 weeks of initiation. These symptoms are non-specific; however, being a delayed-type immune reaction, a patch test can easily help to distinguish hypersensitivity reactions (73). The relevant susceptible gene HLA-B^*^57:01 was found in white and black population (40, 41), especially white (74), and also Hispanic (42) and Indian children (43). A study in Hong Kong found a 0.5% positive rate with HLA-B^*^57:01 in HIV-positive patients of Han Chinese, thus suggesting that screening in this population is unnecessary (75) (Table 1).

Nevirapine is a nun-nucleoside reverse transcriptase inhibitor (NNRTI), and is also a medication treating HIV infection. The association of susceptible gene was first found with HLA-DRB1^*^01:01 in nevirapine-induced HSR in Western Australian (49). However, this gene was later proven more associated with hepatotoxicity rather than cADRs in Caucasian (46). Other susceptible gene found in nevirapine-induced hepatotoxicity were HLA-Cw^*^08 in Japanese (29), HLA-Cw^*^04 in Han Chinese (76), and HLA-DQB1^*^05 in Caucasian (46). Both HLA-Cw^*^08 and HLA-Cw^*^04 were found relevant to nevirapine-induced HSR and hepatoxicity. Relationships between HLA-Cw^*^08 and HSR were found in Sardinian (47) and Japanese (29). HLA-Cw^*^04 was found commonly in nevirapine-induced cADRs in Han Chinese, Thai, Spain, African and Caucasian, especially African and Asian (46). *CYP2B6* was also participated due to affecting delayed plasma clearance of nevirapine (46). Besides, HLA-B^*^35:05 was found to be predictable with nevirapine-induced skin rash in Thai (44), whereas the same relationship was found weak in Han Chinese and Caucasian (46). In addition to HSR, SJS/TEN was found in patients using nevirapine, associated with HLA-C^*^04:01 in African (45), and recently, the association between genetic variations in CCHCR1 and nevirapine-induced HSR was also found in Thai (77). GWAS approach was used in both studies (Table 1).

Raltegravir is an integrase inhibitor that is also used in the treatment of HIV infection, and HLA-B^*^53:01 was found strongly associated with raltegravir-induced DRESS in Africans (50) (Table 1).

### Genetic Susceptibility to Cold Medicine Induced Stevens–Johnson Syndrome and Toxic Epidermal Necrolysis

The term cold medicine comprises non-steroidal anti-inflammatory drugs and other multi-ingredient medications. Due to the high prevalence of cold and the use of cold medicine, SJS/TEN developed after patients using cold medicines is not uncommon, and a series of susceptible genes had been reported to be related to cold medicine-induced SJS/TEN, especially with severe ocular complications. The association of HLA-A^*^02:06 and HLA-B^*^44:03 was found to be associated with cold medicine-induced SJS/TEN in Japanese (55), and the same susceptible genes were verified in patients of other populations, such as HLA-B^*^44:03 in Indian and Brazilian, and HLA^*^02:06 in Korean (56). Besides, HLA-C^*^03:04 was also reported to be associated with cold medicine-induced SJS/TEN in Korean (57). Furthermore, HLA-A^*^66:01 and HLA-C^*^12:03 was found related in Brazilian (58) and HLA-B^*^44:03 and HLA-C^*^07:01 found in Thai (59). In summary, HLA-B^*^44:03 seems to be a cross-ethnic susceptible gene with a strong association in Japanese, Brazilian, Indian, and Thai, whereas HLA-A^*^02:06, HLA-B^*^44:03, toll-like receptor 3 (TLR3), prostaglandin-E receptor 3 (PTGER3), and IKZF1, by using GWAS approach, were identified as primary association to cold medicine-induced SJS/TEN with severe ocular involvement in Japanese (55, 60, 61, 78) (Table 1).

### Genetic Susceptibility to Allopurinol-Induced Severe Cutaneous Adverse Reactions

By inhibiting xanthine oxidase and then reducing the synthesis of uric acid, allopurinol is a first-line drug used to treat hyperuricemia and gout and also remains to be one of the leading causes of SCARs. HLA-B^*^5801 was first found as an important genet marker of allopurinol-induced SCARs in Han Chinese (64). The same associations were validated in Thai (62), Japanese (63), Korean (65), and Caucasians (34). However, the pharmacogenomic associations varied from different ethnic populations, 50–60% in Caucasians and Japanese, and 80–100% in Korea, Thai, and Han Chinese (79). Other non-genetic factors may also contribute to allopurinol-induced SCARs, especially impaired renal function, cardiovascular diseases, and higher drug initiating dosages (80) (Table 1).

### Genetic Susceptibility to Methazolamide-Induced Stevens–Johnson Syndrome and Toxic Epidermal Necrolysis

Carbonic anhydrase inhibitor methazolamide is a medication applied in lowering intraocular pressure, for example, as a patient with glaucoma. Despite the low frequency, methazolamide may potentially induce SJS/TEN. Strong association of HLA-B^*^59:01 and methazolamide-induced SJS/TEN in Korean and Japanese had been reported (66) and also in Han Chinese (67) (Table 1).

### Jackpot Theory and Importance of Specific T Cell Receptor for the Development of Severe Cutaneous Adverse Reactions

Although previous studies proved the association of specific HLA genotypes with SCARs, the positive predictive values were mostly low (81). Our previous study showed CBZ-specific T cells expressing specific TCR from peripheral blood mononuclear cells and causing activation of granulysin release in HLA-B^*^15:02-positive CBZ-induced SJS/TEN but was not found in CBZ-tolerant individuals or other drug-related SJS/TEN (82). Similarly, preferential usage of TCRβ variable gene and clonal expansion of specific third complementarity-determining region (CDR3) was identified in blister cells of patients with allopurinol-induced SJS/TEN (83). More recently, our further study showed a public αβTCR was further identified from CTLs of patients with CBZ-induced SJS/TEN, with a paired TCRβ CDR3 clonotype “ASSLAGELF” and TCRα CDR3 clonotype “VFDNTDKLI,” which react to CBZ and its structural analogs by its structural analogs and bind affinity and mediate immune response (84). Importantly, the specific CDR3 clonotype was present in CTLs of CBZ-induced SJS/TEN of patients from different populations (Chinese or Europeans) with or without the HLA-B^*^1502 genotype. The finding of clonotype-specific T cell and drug-specific public TCR in patients illustrated the essential molecular role of shared and restricted TCR that interacted with specific HLA and drugs for the development of SCARs (Jackpot theory).

### Pharmacogenomic Test for Prevention of Severe Cutaneous Adverse Reactions

The burden of developing SCARs was life-threatening. Not only public health but also medical resources were affected. A team in Singapore observed higher laboratory costs in the residency of patients with ADRs (85).

Considering the mortality rate and economic burden of SCARs, an easy and effective method for the prevention of SCARs would be greatly needed. The development of a strong correlation of preemptive genetic tests for high-risk medications related to SCARs is therefore very helpful. By screening relevant genes before using susceptible culprit drugs, we should be able to prevent the occurrence of SCARs and the consequent cost for sequalae.

Genetic testing of HLA-B^*^15:02 before CBZ was warned by The Food and Drug Administration (FDA) of the United States, Taiwan, and similar agencies of other countries (86), and genetic testing of HLA-B^*^58:01 before allopurinol initiation was warned by the FDA of Taiwan or other Asian countries (Table 1). Recommendations of relative gene testing before culprit drugs in case of developing SCARs were noticed in various studies (87). The first prospective screening test was applied in patients with HIV infection needed for abacavir and demonstrated a significantly lower incidence in the HLA-B^*^57:01 screening group (74). For proof of concept and clinical implementation, the Taiwan SJS Consortium had conducted a clinical trial to prevent CBZ-induced SCARs by genotyping DNA with HLA-B^*^15:02 allele of 4,877 candidates before CBZ therapy and found no SJS/TEN with negative subjects (88). The other one of the most commonly used drugs and cause of SCARs in Taiwan was allopurinol. Prospective screening with HLA-B^*^58:01 enrolled 2,926 patients before initiation of allopurinol in Taiwan, and the result showed none of the negative subjects developed SCAR (89). Recently, a prospective study of 1,539 patients diagnosed with leprosy underwent HLA-B^*^13:01 genotyping before using dapsone in China, and the result showed no SCARs developed in non-carrier (90) (Table 1).

### Implementation of Precision-Based Use of Antiepileptic Drug Therapy by Screening Multiple Risk Alleles Related to Aromatic Antiepileptic Drugs for Prevention of Severe Cutaneous Adverse Reactions

With a similar aromatic ring structure, the first line AEDs, such as CBZ, OXC, LTG, PHT, and PB, have potential and share similar risk alleles predisposing to SCARs. For consideration of efficacy and safety for clinical selection of AEDs, a piece of pharmacogenomics information is important for decision making for patients with epileptic or neurologic disorders. Therefore, pharmacogenomic panel testing for multiple risk alleles related to all AEDs has its clinical necessity and demand; for clinical implementation and to evaluate the feasibility and efficacy of a pharmacogenomic panel of AEDs, we have developed a rapid testing panel of multiple risk alleles, including HLA-B^*^15:02, CYP2C9^*^3, HLA-B^*^13:01, HLA-B^*^51:01, and HLA-A^*^31:01, which are strongly related to aromatic AED-induced SCARs (Table 2) for decision making before initiating AEDs. A clinical trial has started since 2018 in Taiwan and China of multiple medical centers, by enrolling patients with need of antiepileptic drugs without history of using, and performing the testing panel by quantitative polymerase chain reaction (qPCR). With avoiding relevant drugs with positive susceptible genes, a preliminary evaluation of 231 patients with negative corresponding risk alleles received aromatic AEDs therapy, no SCARs have been observed, although five patients developed milder maculopapular eruption or itchy skin lesions.

**Table 2:** Pharmacogenomic panel linked to AED-related SCARs in Asians.

	**Risk alleles**	**Sensitivity (%)**	**Specificity (%)**	**Reference**
CBZ-SCARs	HLA-B*15:02 HLA-A*31:01	74.6	90.3	(31)
OXC-SCARs	HLA-B*15:02	70.6	92.1	(23)
PHT-SCARs	HLA-B*15:02 HLA-B*13:01 HLA-B*51:01 CYP2C9*3	71.9	77.7	(37)
LTG-SCARs	HLA-B*15:02	29.4	89.7	(69)

## Conclusions

SCARs are rare but life-threatening. The findings of the relationship with susceptible genes and drugs lead to clinical applications of prevention. By screening the known relevant genes before prescribing potentially culprit drugs in corresponding ethnicities would be an effective method to avoid the development of SCARs.

## Author Contributions

S-CY, M-YL, Z-YZ, X-YJ, MH, Y-FZ, C-BC, and W-HC wrote the original draft. X-YJ, MH, and Y-FZ provided the resources. S-CY, M-YL, Z-YZ, C-BC, and W-HC reviewed and edited the draft. C-BC and W-HC conceptualized the review and acquired funding. All authors contributed to the article and approved the submitted version.

## Conflict of Interest

The authors declare that the research was conducted in the absence of any commercial or financial relationships that could be construed as a potential conflict of interest.

## References

[1] Chung WH Wang CW Dao RL. Severe cutaneous adverse drug reactions. J Dermatol. (2016) 43:758–66. 10.1111/1346-8138.13430 27154258

[2] Sunaga Y Kurosawa M Ochiai H Watanabe H Sueki H Azukizawa H. The nationwide epidemiological survey of Stevens-Johnson syndrome and toxic epidermal necrolysis in Japan, 2016-2018. J Dermatol Sci. (2020) 100:175–82. 10.1016/j.jdermsci.2020.09.009 33046331

[3] Yang MS Lee JY Kim J Kim GW Kim BK Kim JY. Searching for the culprit drugs for Stevens-Johnson syndrome and toxic epidermal necrolysis from a nationwide claim database in Korea. J Allergy Clin Immunol Pract. (2020) 8:690–5 e692. 10.1016/j.jaip.2019.09.032 31614216

[4] Yang SC Hu S Zhang SZ Huang JW Zhang J Ji C. The epidemiology of Stevens-Johnson syndrome and toxic epidermal necrolysis in China. J Immunol Res. (2018) 2018:4320195. 10.1155/2018/4154507 29607330 PMC5828103

[5] Dodiuk-Gad RP Chung WH Valeyrie-Allanore L Shear NH. Stevens-Johnson syndrome and toxic epidermal necrolysis: an update. Am J Clin Dermatol. (2015) 16:475–93. 10.1007/s40257-015-0158-0 26481651

[6] Wedi B. Definitions and mechanisms of drug hypersensitivity. Expert Rev Clin Pharmacol. (2010) 3:539–51. 10.1586/ecp.10.32 22111682

[7] Chung WH Hung SI. Recent advances in the genetics and immunology of Stevens-Johnson syndrome and toxic epidermal necrosis. J Dermatol Sci. (2012) 66:190–6. 10.1016/j.jdermsci.2012.04.002 22541332

[8] Chung WH Hung SI Yang JY Su SC Huang SP Wei CY. Granulysin is a key mediator for disseminated keratinocyte death in Stevens-Johnson syndrome and toxic epidermal necrolysis. Nat Med. (2008) 14:1343–50. 10.1038/nm.1884 19029983

[9] Kardaun SH Sekula P Valeyrie-Allanore L Liss Y Chu CY Creamer D. Drug reaction with eosinophilia and systemic symptoms (DRESS): an original multisystem adverse drug reaction. Results from the prospective RegiSCAR study. Br J Dermatol. (2013) 169:1071–80. 10.1111/bjd.12501 23855313

[10] Komatsu-Fujii T Kaneko S Chinuki Y Suyama Y Ohta M Niihara H. Serum TARC levels are strongly correlated with blood eosinophil count in patients with drug eruptions. Allergol Int. (2017) 66:116–22. 10.1016/j.alit.2016.06.003 27497618

[11] Roujeau JC Bracq C Huyn NT Chaussalet E Raffin C Duedari N. HLA phenotypes and bullous cutaneous reactions to drugs. Tissue Antigens. (1986) 28:251–4. 10.1111/j.1399-0039.1986.tb00491.x 3544335

[12] Roujeau JC Huynh TN Bracq C Guillaume JC Revuz J Touraine R. Genetic susceptibility to toxic epidermal necrolysis. Arch Dermatol. (1987) 123:1171–3. 3477129

[13] Chang CJ Chen CB Hung SI Ji C Chung WH. Pharmacogenetic testing for prevention of severe cutaneous adverse drug reactions. Front Pharmacol. (2020) 11:969. 10.3389/fphar.2020.00969 32714190 PMC7346738

[14] Aihara M. Pharmacogenetics of cutaneous adverse drug reactions. J Dermatol. (2011) 38:246–54. 10.1111/j.1346-8138.2010.01196.x 21342226

[15] Tong H Phan NVT Nguyen TT Nguyen DV Vo NS Le L. Review on databases and bioinformatic approaches on pharmacogenomics of adverse drug reactions. Pharmgenomics Pers Med. (2021) 14:61–75. 10.2147/PGPM.S290781 33469342 PMC7812041

[16] Ueta M. Results of detailed investigations into stevens-johnson syndrome with severe ocular complications. Invest Ophthalmol Vis Sci. (2018) 59:DES183–DES191. 10.1167/iovs.17-23537 30481825

[17] Wang CW Tassaneeyakul W Chen CB Chen WT Teng YC Huang CY. Whole genome sequencing identifies genetic variants associated with co-trimoxazole hypersensitivity in Asians. J Allergy Clin Immunol. (2021). 147:1402–12. 10.1016/j.jaci.2020.08.003 32791162

[18] Chung WH Hung SI Hong HS Hsih MS Yang LC Ho HC. Medical genetics: a marker for Stevens-Johnson syndrome. Nature. (2004) 428:486. 10.1038/428486a 15057820

[19] Tassaneeyakul W Tiamkao S Jantararoungtong T Chen P Lin SY Chen WH. Association between HLA-B^*^1502 and carbamazepine-induced severe cutaneous adverse drug reactions in a Thai population. Epilepsia. (2010) 51:926–30. 10.1111/j.1528-1167.2010.02533.x 20345939

[20] Chang CC Too CL Murad S Hussein SH. Association of HLA-B^*^1502 allele with carbamazepine-induced toxic epidermal necrolysis and Stevens-Johnson syndrome in the multi-ethnic Malaysian population. Int J Dermatol. (2011) 50:221–4. 10.1111/j.1365-4632.2010.04745.x 21244392

[21] Mehta TY Prajapati LM Mittal B Joshi CG Sheth JJ Patel DB. Association of HLA-B^*^1502 allele and carbamazepine-induced Stevens-Johnson syndrome among Indians. Indian J Dermatol Venereol Leprol. (2009) 75:579–82. 10.4103/0378-6323.57718 19915237

[22] Man CB Kwan P Baum L Yu E Lau KM Cheng AS. Association between HLA-B^*^1502 allele and antiepileptic drug-induced cutaneous reactions in Han Chinese. Epilepsia. (2007) 48:1015–8. 10.1111/j.1528-1167.2007.01022.x 17509004

[23] Chen CB Hsiao YH Wu T Hsih MS Tassaneeyakul W Jorns TP. Risk and association of HLA with oxcarbazepine-induced cutaneous adverse reactions in Asians. Neurology. (2017) 88:78–86. 10.1212/WNL.0000000000003453 27913699

[24] Chang CC Ng CC Too CL Choon SE Lee CK Chung WH. Association of HLA-B^*^15:13 and HLA-B^*^15:02 with phenytoin-induced severe cutaneous adverse reactions in a Malay population. Pharmacogenomics J. (2017) 17:170–3. 10.1038/tpj.2016.10 26927288

[25] Hung SI Chung WH Liu ZS Chen CH Hsih MS Hui RC. Common risk allele in aromatic antiepileptic-drug induced Stevens-Johnson syndrome and toxic epidermal necrolysis in Han Chinese. Pharmacogenomics. (2010) 11:349–56. 10.2217/pgs.09.162 20235791

[26] Locharernkul C Loplumlert J Limotai C Korkij W Desudchit T Tongkobpetch S. Carbamazepine and phenytoin induced Stevens-Johnson syndrome is associated with HLA-B^*^1502 allele in Thai population. Epilepsia. (2008) 49:2087–91. 10.1111/j.1528-1167.2008.01719.x 18637831

[27] Kaniwa N Saito Y Aihara M Matsunaga K Tohkin M Kurose K. HLA-B^*^1511 is a risk factor for carbamazepine-induced Stevens-Johnson syndrome and toxic epidermal necrolysis in Japanese patients. Epilepsia. (2010) 51:2461–5. 10.1111/j.1528-1167.2010.02766.x 21204807

[28] Kim SH Lee KW Song WJ Kim SH Jee YK Lee SM. Carbamazepine-induced severe cutaneous adverse reactions and HLA genotypes in Koreans. Epilepsy Res. (2011) 97:190–7. 10.1016/j.eplepsyres.2011.08.010 21917426

[29] Jaruthamsophon K Tipmanee V Sangiemchoey A Sukasem C Limprasert P. HLA-B*15:21 and carbamazepine-induced Stevens-Johnson syndrome: pooled-data and in silico analysis. Sci Rep. (2017) 7:45553. 10.1038/srep45553 28358139 PMC5372085

[30] Mockenhaupt M Wang CW Hung SI Sekula P Schmidt AH Pan RY. HLA-B^*^57:01 confers genetic susceptibility to carbamazepine-induced SJS/TEN in Europeans. Allergy. (2019) 74:2227–30. 10.1111/all.13821 30972788

[31] Genin E Chen DP Hung SI Sekula P Schumacher M Chang PY. HLA-A^*^31:01 and different types of carbamazepine-induced severe cutaneous adverse reactions: an international study and meta-analysis. Pharmacogenomics J. (2014) 14:281–8. 10.1038/tpj.2013.40 24322785

[32] McCormack M Alfirevic A Bourgeois S Farrell JJ Kasperaviciute D Carrington M. HLA-A^*^3101 and carbamazepine-induced hypersensitivity reactions in Europeans. N Engl J Med. (2011) 364:1134–43. 10.1056/NEJMoa1013297 21428769 PMC3113609

[33] Mushiroda T Takahashi Y Onuma T Yamamoto Y Kamei T Hoshida T. Association of HLA-A^*^31:01 screening with the incidence of carbamazepine-induced cutaneous adverse reactions in a Japanese population. JAMA Neurol. (2018) 75:842–9. 10.1001/jamaneurol.2018.0278 29610831 PMC6145764

[34] Lonjou C Borot N Sekula P Ledger N Thomas L Halevy S. A European study of HLA-B in Stevens-Johnson syndrome and toxic epidermal necrolysis related to five high-risk drugs. Pharmacogenet Genom. (2008) 18:99–107. 10.1097/FPC.0b013e3282f3ef9c 18192896

[35] Chung WH Chang WC Lee YS Wu YY Yang CH Ho HC. Genetic variants associated with phenytoin-related severe cutaneous adverse reactions. JAMA. (2014) 312:525–34. 10.1001/jama.2014.7859 25096692

[36] Kaniwa N Sugiyama E Saito Y Kurose K Maekawa K Hasegawa R. Specific HLA types are associated with antiepileptic drug-induced Stevens-Johnson syndrome and toxic epidermal necrolysis in Japanese subjects. Pharmacogenomics. (2013) 14:1821–31. 10.2217/pgs.13.180 24236482

[37] Su SC Chen CB Chang WC Wang CW Fan WL Lu LY. HLA Alleles and CYP2C9*3 as predictors of phenytoin hypersensitivity in East Asians. Clin Pharmacol Ther. (2019) 105:476–85. 10.1002/cpt.1190 30270535

[38] Rutkowski K Taylor C Wagner A. HLA B62 as a possible risk factor for drug reaction with eosinophilia and systemic symptoms to piperacillin/tazobactam. J Allergy Clin Immunol Pract. (2017) 5:829–30. 10.1016/j.jaip.2016.10.008 27914818

[39] Konvinse KC Trubiano JA Pavlos R James I Shaffer CM Bejan CA. HLA-A^*^32:01 is strongly associated with vancomycin-induced drug reaction with eosinophilia and systemic symptoms. J Allergy Clin Immunol. (2019) 144:183–92. 10.1016/j.jaci.2019.01.045 30776417 PMC6612297

[40] Hetherington S Hughes AR Mosteller M Shortino D Baker KL Spreen W. Genetic variations in HLA-B region and hypersensitivity reactions to abacavir. Lancet. (2002) 359:1121–2. 10.1016/S0140-6736(02)08158-8 11943262

[41] Mallal S Nolan D Witt C Masel G Martin AM Moore C. Association between presence of HLA-B^*^5701, HLA-DR7, and HLA-DQ3 and hypersensitivity to HIV-1 reverse-transcriptase inhibitor abacavir. Lancet. (2002) 359:727–32. 10.1016/S0140-6736(02)07873-X 11888582

[42] Hughes AR Mosteller M Bansal AT Davies K Haneline SA Lai EH. Association of genetic variations in HLA-B region with hypersensitivity to abacavir in some, but not all, populations. Pharmacogenomics. (2004) 5:203–11. 10.1517/phgs.5.2.203.27481 15016610

[43] Manglani MV Gabhale YR Lala MM Sekhar R More D. HLA- B^*^5701 allele in HIV-infected indian children and its association with abacavir hypersensitivity. Indian Pediatr. (2018) 55:140–1. 10.1007/s13312-018-1248-x 29242412

[44] Chantarangsu S Mushiroda T Mahasirimongkol S Kiertiburanakul S Sungkanuparph S Manosuthi W. HLA-B^*^3505 allele is a strong predictor for nevirapine-induced skin adverse drug reactions in HIV-infected Thai patients. Pharmacogenet Genomics. (2009) 19:139–46. 10.1097/FPC.0b013e32831d0faf 19104471

[45] Carr DF Bourgeois S Chaponda M Takeshita LY Morris AP Castro EM. (2017). Genome-wide association study of nevirapine hypersensitivity in a sub-Saharan African HIV-infected population. J Antimicrob Chemother. 72:1152–62. 10.1093/jac/dkw545 28062682 PMC5400091

[46] Yuan J Guo S Hall D Cammett AM Jayadev S Distel M. Toxicogenomics of nevirapine-associated cutaneous and hepatic adverse events among populations of African, Asian, and European descent. AIDS. (2011) 25:1271–80. 10.1097/QAD.0b013e32834779df 21505298 PMC3387531

[47] Littera R Carcassi C Masala A Piano P Serra P Ortu F. HLA-dependent hypersensitivity to nevirapine in Sardinian HIV patients. AIDS. (2006) 20:1621–6. 10.1097/01.aids.0000238408.82947.09 16868443

[48] Gatanaga H Yazaki H Tanuma J Honda M Genka I Teruya K. HLA-Cw8 primarily associated with hypersensitivity to nevirapine. AIDS. (2007) 21:264–5. 10.1097/QAD.0b013e32801199d9 17197830

[49] Martin AM Nolan D James I Cameron P Keller J Moore C. Predisposition to nevirapine hypersensitivity associated with HLA-DRB1^*^0101 and abrogated by low CD4 T-cell counts. AIDS. (2005) 19:97–9. 10.1097/00002030-200501030-00014 15627041

[50] Thomas M Hopkins C Duffy E Lee D Loulergue P Ripamonti D. Association of the HLA-B^*^53:01 allele with drug reaction with eosinophilia and systemic symptoms (DRESS) syndrome during treatment of HIV infection with Raltegravir. Clin Infect Dis. (2017) 64:1198–203. 10.1093/cid/cix096 28369189 PMC5399947

[51] Zhang FR Liu H Irwanto A Fu XA Li Y Yu GQ. HLA-B^*^13:01 and the dapsone hypersensitivity syndrome. N Engl J Med. (2013) 369:1620–8. 10.1056/NEJMoa1213096 24152261

[52] Park HJ Park JW Kim SH Choi SY Kim HK Jung CG. The HLA-B^*^13:01 and the dapsone hypersensitivity syndrome in Korean and Asian populations: genotype- and meta-analyses. Expert Opin Drug Saf. (2020) 19:1349–56. 10.1080/14740338.2020.1796965 32700588

[53] Krismawati H Irwanto A Pongtiku A Irwan ID Maladan Y Sitanggang YA. Validation study of HLA-B^*^13:01 as a biomarker of dapsone hypersensitivity syndrome in leprosy patients in Indonesia. PLoS Negl Trop Dis. (2020) 14:e0008746. 10.1371/journal.pntd.0008746 33064728 PMC7592909

[54] Tempark T Satapornpong P Rerknimitr P Nakkam N Saksit N Wattanakrai P. Dapsone-induced severe cutaneous adverse drug reactions are strongly linked with HLA-B^*^13: 01 allele in the Thai population. Pharmacogenet Genomics. (2017) 27:429–37. 10.1097/FPC.0000000000000306 28885988

[55] Ueta M Kaniwa N Sotozono C Tokunaga K Saito Y Sawai H. Independent strong association of HLA-A^*^02:06 and HLA-B^*^44:03 with cold medicine-related Stevens-Johnson syndrome with severe mucosal involvement. Sci Rep. (2014) 4:4862. 10.1038/srep04862 24781922 PMC5381277

[56] Ueta M Kannabiran C Wakamatsu TH Kim MK Yoon KC Seo KY. Trans-ethnic study confirmed independent associations of HLA-A^*^02:06 and HLA-B^*^44:03 with cold medicine-related Stevens-Johnson syndrome with severe ocular surface complications. Sci Rep. (2014) 4:5981. 10.1038/srep05981 25099678 PMC4124463

[57] Jun I Rim JH Kim MK Yoon KC Joo CK Kinoshita S. Association of human antigen class I genes with cold medicine-related Stevens-Johnson syndrome with severe ocular complications in a Korean population. Br J Ophthalmol. (2019) 103:573–6. 10.1136/bjophthalmol-2018-313263 30705045

[58] Wakamatsu TH Ueta M Tokunaga K Okada Y Loureiro RR Costa KA. Human leukocyte antigen class i genes associated with Stevens-Johnson syndrome and severe ocular complications following use of cold medicine in a Brazilian Population. JAMA Ophthalmol. (2017) 135:355–60. 10.1001/jamaophthalmol.2017.0074 28278336 PMC5470496

[59] Jongkhajornpong P Lekhanont K Pisuchpen P Chantaren P Puangsricharern V Prabhasawat P. Association between HLA-B^*^44:03-HLA-C^*^07:01 haplotype and cold medicine-related Stevens-Johnson syndrome with severe ocular complications in Thailand. Br J Ophthalmol. (2018) 102:1303–7. 10.1136/bjophthalmol-2017-311823 29706602

[60] Ueta M Sotozono C Nakano M Taniguchi T Yagi T Tokuda Y. Association between prostaglandin E receptor 3 polymorphisms and Stevens-Johnson syndrome identified by means of a genome-wide association study. J Allergy Clin Immunol. (2010) 126:1218–25.e1210. 10.1016/j.jaci.2010.08.007 20947153

[61] Ueta M Sawai H Sotozono C Hitomi Y Kaniwa N Kim MK. (2015). IKZF1, a new susceptibility gene for cold medicine-related Stevens-Johnson syndrome/toxic epidermal necrolysis with severe mucosal involvement. J Allergy Clin Immunol. 135:1538–45.e1517. 10.1016/j.jaci.2014.12.1916 25672763

[62] Tassaneeyakul W Jantararoungtong T Chen P Lin PY Tiamkao S Khunarkornsiri U. Strong association between HLA-B^*^5801 and allopurinol-induced Stevens-Johnson syndrome and toxic epidermal necrolysis in a Thai population. Pharmacogenet Genomics. (2009) 19:704–9. 10.1097/FPC.0b013e328330a3b8 19696695

[63] Kaniwa N Saito Y Aihara M Matsunaga K Tohkin M Kurose K. HLA-B locus in Japanese patients with anti-epileptics and allopurinol-related Stevens-Johnson syndrome and toxic epidermal necrolysis. Pharmacogenomics. (2008) 9:1617–22. 10.2217/14622416.9.11.1617 19018717

[64] Hung SI Chung WH Liou LB Chu CC Lin M Huang HP. HLA-B^*^5801 allele as a genetic marker for severe cutaneous adverse reactions caused by allopurinol. Proc Natl Acad Sci USA. (2005) 102:4134–9. 10.1073/pnas.0409500102 15743917 PMC554812

[65] Kang HR Jee YK Kim YS Lee CH Jung JW Kim SH. Positive and negative associations of HLA class I alleles with allopurinol-induced SCARs in Koreans. Pharmacogenet Genomics. (2011) 21:303–7. 10.1097/FPC.0b013e32834282b8 21301380

[66] Kim SH Kim M Lee KW Kim SH Kang HR Park HW. HLA-B^*^5901 is strongly associated with methazolamide-induced Stevens-Johnson syndrome/toxic epidermal necrolysis. Pharmacogenomics. (2010) 11:879–84. 10.2217/pgs.10.54 20504258

[67] Yang F Xuan J Chen J Zhong H Luo H Zhou P. HLA-B^*^59:01: a marker for Stevens-Johnson syndrome/toxic epidermal necrolysis caused by methazolamide in Han Chinese. Pharmacogenomics J. (2016) 16:83–7. 10.1038/tpj.2015.25 25918017

[68] Wang W Hu FY Wu XT An DM Yan B Zhou D. Genetic predictors of Stevens-Johnson syndrome and toxic epidermal necrolysis induced by aromatic antiepileptic drugs among the Chinese Han population. Epilepsy Behav. (2014) 37:16–9. 10.1016/j.yebeh.2014.05.025 24949577

[69] Cheung YK Cheng SH Chan EJ Lo SV Ng MH Kwan P. HLA-B alleles associated with severe cutaneous reactions to antiepileptic drugs in Han Chinese. Epilepsia. (2013) 54:1307–14. 10.1111/epi.12217 23692434

[70] Ozeki T Mushiroda T Yowang A Takahashi A Kubo M Shirakata Y. Genome-wide association study identifies HLA-A*3101 allele as a genetic risk factor for carbamazepine-induced cutaneous adverse drug reactions in Japanese population. Hum Mol Genet. (2011) 20:1034–41. 10.1093/hmg/ddq537 21149285

[71] Blumenthal KG Peter JG Trubiano JA Phillips EJ. Antibiotic allergy. Lancet. (2019) 393:183–98. 10.1016/S0140-6736(18)32218-9 30558872 PMC6563335

[72] Norton AE Konvinse K Phillips EJ Broyles AD. Antibiotic allergy in pediatrics. Pediatrics. (2018) 141:e20172497. 10.1542/peds.2017-2497 29700201 PMC5914499

[73] Rodriguez-Novoa S Soriano V. Current trends in screening across ethnicities for hypersensitivity to abacavir. Pharmacogenomics. (2008) 9:1531–41. 10.2217/14622416.9.10.1531 18855539

[74] Mallal S Phillips E Carosi G Molina JM Workman C Tomazic J. HLA-B^*^5701 screening for hypersensitivity to abacavir. N Engl J Med. (2008) 358:568–79. 10.1056/NEJMoa0706135 18256392

[75] To SW Chen JH Wong KH Chan KC Tsang OT Yam WC. HLA-B^*^5701 genetic screening among HIV-1 infected patients in Hong Kong: is this a practical approach in Han-Chinese? Int J STD AIDS. (2013) 24:50–2. 10.1258/ijsa.2012.012102 23512513

[76] Gao S Gui XE Liang K Liu Z Hu J Dong B. HLA-dependent hypersensitivity reaction to nevirapine in Chinese Han HIV-infected patients. AIDS Res Hum Retroviruses. (2012) 28:540–3. 10.1089/aid.2011.0107 21902584

[77] Chantarangsu S Mushiroda T Mahasirimongkol S Kiertiburanakul S Sungkanuparph S Manosuthi W. Genome-wide association study identifies variations in 6p21.3 associated with nevirapine-induced rash. Clin Infect Dis. (2011) 53:341–8. 10.1093/cid/cir403 21810746

[78] Nakatani K Ueta M Khor SS Hitomi Y Okudaira Y Masuya A. Identification of HLA-A^*^02:06:01 as the primary disease susceptibility HLA allele in cold medicine-related Stevens-Johnson syndrome with severe ocular complications by high-resolution NGS-based HLA typing. Sci Rep. (2019) 9:16240. 10.1038/s41598-019-52619-2 31700100 PMC6838058

[79] Yu KH Yu CY Fang YF. Diagnostic utility of HLA-B^*^5801 screening in severe allopurinol hypersensitivity syndrome: an updated systematic review and meta-analysis. Int J Rheum Dis. (2017) 20:1057–71. 10.1111/1756-185X.13143 28857441

[80] Wang CW Dao RL Chung WH. Immunopathogenesis and risk factors for allopurinol severe cutaneous adverse reactions. Curr Opin Allergy Clin Immunol. (2016) 16:339–45. 10.1097/ACI.0000000000000286 27362322

[81] Pan RY Dao RL Hung SI Chung WH. Pharmacogenomic advances in the prediction and prevention of cutaneous idiosyncratic drug reactions. Clin Pharmacol Ther. (2017) 102:86–97. 10.1002/cpt.683 28295240

[82] Ko TM Chung WH Wei CY Shih HY Chen JK Lin CH. Shared and restricted T-cell receptor use is crucial for carbamazepine-induced Stevens-Johnson syndrome. J Allergy Clin Immunol. (2011) 128:1266–76 e1211. 10.1016/j.jaci.2011.08.013 21924464

[83] Chung WH Pan RY Chu MT Chin SW Huang YL Wang WC. Oxypurinol-specific T cells possess preferential TCR clonotypes and express granulysin in allopurinol-induced severe cutaneous adverse reactions. J Invest Dermatol. (2015) 135:2237–48. 10.1038/jid.2015.165 25946710

[84] Pan RY Chu MT Wang CW Lee YS Lemonnier F Michels AW. Identification of drug-specific public TCR driving severe cutaneous adverse reactions. Nat Commun. (2019) 10:3569. 10.1038/s41467-019-11396-2 31395875 PMC6687717

[85] Chan SL Ng HY Sung C Chan A Winther MD Brunham LR. Economic burden of adverse drug reactions and potential for pharmacogenomic testing in Singaporean adults. Pharmacogenomics J. (2019) 19:401–410. 10.1038/s41397-018-0053-1 30250149

[86] Ferrell PB Jr McLeod HL. Carbamazepine, HLA-B^*^1502 and risk of Stevens-Johnson syndrome and toxic epidermal necrolysis: US FDA recommendations. Pharmacogenomics. (2008) 9:1543–6. 10.2217/14622416.9.10.1543 18855540 PMC2586963

[87] Khor AH Lim KS Tan CT Kwan Z Tan WC Wu DB. HLA-A^*^31: 01 and HLA-B^*^15:02 association with Stevens-Johnson syndrome and toxic epidermal necrolysis to carbamazepine in a multiethnic Malaysian population. Pharmacogenet Genomics. (2017) 27:275–8. 10.1097/FPC.0000000000000287 28570299

[88] Chen P Lin JJ Lu CS Ong CT Hsieh PF Yang CC. Carbamazepine-induced toxic effects and HLA-B^*^1502 screening in Taiwan. N Engl J Med. (2011) 364:1126–33. 10.1056/NEJMoa1009717 21428768

[89] Ko TM Tsai CY Chen SY Chen KS Yu KH Chu CS. Use of HLA-B^*^58:01 genotyping to prevent allopurinol induced severe cutaneous adverse reactions in Taiwan: national prospective cohort study. BMJ. (2015) 351:h4848. 10.1136/bmj.h4848 26399967 PMC4579807

[90] Liu H Wang Z Bao F Wang C Sun L Zhang H. Evaluation of prospective HLA-B^*^13:01 screening to prevent dapsone hypersensitivity syndrome in patients with leprosy. JAMA Dermatol. (2019) 155:666–72. 10.1001/jamadermatol.2018.5360 30916737 PMC6563542

